# High glucose induces formation of tau hyperphosphorylation via Cav-1-mTOR pathway: A potential molecular mechanism for diabetes-induced cognitive dysfunction

**DOI:** 10.18632/oncotarget.17257

**Published:** 2017-04-19

**Authors:** Jing Wu, Shan-Lei Zhou, Lin-Hua Pi, Xia-Jie Shi, Ling-Ran Ma, Zi Chen, Min-Li Qu, Xin Li, Sheng-Dan Nie, Duan-Fang Liao, Jin-Jing Pei, Shan Wang

**Affiliations:** ^1^ Department of Endocrinology, Xiang-Ya Hospital, Central South University, Changsha, Hunan, China; ^2^ Department of Pharmaceutical Engineering, College of Chemistry and Chemical Engineering, Central South University, Changsha, Hunan, China; ^3^ Institute of Clinical Medicine, People's Hospital of Hunan Province, The First Affiliated Hospital of Hunan Normal University, Changsha, Hunan, China; ^4^ Division of Stem Cell Regulation and Application, School of Pharmacy, Hunan University of Traditional Chinese Medicine, Changsha, Hunan, China; ^5^ KI-Alzheimer's Disease Research Center, Karolinska Institutet, Novum, Stockholm, Sweden; ^6^ Department of Neurology, Xuan Wu Hospital, Capital Medical University, Xicheng, Beijing, China; ^7^ Center of Alzheimer's Disease, Beijing Institute for Brain Disorders, Beijing, China

**Keywords:** diabetes mellitus, cognitive dysfunction, caveolin-1, tau hyperphosphorylation, mTOR

## Abstract

The abnormally hyperphosphorylated tau is thought to be implicated in diabetes-associated cognitive deficits. The role of mammalian target of rapamycin (mTOR) / S6 kinase (S6K) signalling in the formation of tau hyperphosphorylation has been previously studied. Caveolin-1 (Cav-1), the essential structure protein of caveolae, promotes neuronal survival and growth, and inhibits glucose metabolism. In this study, we aimed to investigate the role of Cav-1 in the formation of tau hyperphosphorylation under chronic hyperglycemic condition (HGC). Diabetic rats were induced by streptozotocin (STZ). Primary hippocampal neurons with or without molecular intervention such as the transient over-expression or knock-down were subjected to HGC. The obtained experimental samples were analyzed by real time quantitative RT-PCR, Western blot, immunofluorescence or immunohistochemisty. We found: 1) that a chronic HGC directly decreases Cav-1 expression, increases tau phosphorylation and activates mTOR/S6K signalling in the brain neurons of diabetic rats, 2) that overexpression of Cav-1 attenuates tau hyperphosphorylation induced by chronic HGC in primary hippocampal neurons, whereas down-regulation of Cav-1 using Cav-1 siRNA dramatically worsens tau hyperphosphorylation via mTOR/S6K signalling pathway, and 3) that the down-regulation of Cav-1 induced by HGC is independent of mTOR signalling. Our results suggest that tau hyperphosphorylation and the sustained over-activated mTOR signalling under hyperglycemia may be due to the suppression of Cav-1. Therefore, Cav-1 is a potential therapeutic target for diabetes-induced cognitive dysfunction.

## INTRODUCTION

Diabetes mellitus (DM), characterized by hyperglycemia, has adverse effects on the brain of affected subjects, especially on the hippocampus, a brain area particularly susceptible to harmful stimuli [1]. A large number of epidemiological findings have suggested that DM patients are at risk for developing cognitive decline [2, 3]. Experimental studies have found significant cognitive deficits in streptozotocin (STZ)-induced hyperglycemic diabetic rats [4].

Tau is a neuron-enriched phospho-protein, is one of the major microtubule-associated proteins. It normally binds to microtubules, promotes tubulin assembly into microtubules and stabilizes microtubules in neurons [5]. When tau is abnormally hyperphosphorylated, it detaches from microtubules and aggregates into paired helical filaments (PHFs) in neurofibrillary tangles (NFTs), a characteristic neuropathological hallmarker in the brains of patients diagnosed as tauopathies, of which Alzheimer disease (AD) is the most common form [6]. It has been reported that the severity of tau-pathologies strongly correlates with cognitive impairment in AD patients [7]. Similar to AD, DM is also considered as tauopathy-associated disease based on the formation of tau hyperphosphorylation in DM brains [8–10]. Alonso et al. have demonstrated that transfected PC 12 cells with pseudophosphorylated tau caused neurodegeneration [11]. Di J et al. created an inducible pseudophosphorylated tau mouse model to study the effect of conformationally modified tau *in vivo* [12], although rodent tau does not naturally form PHFs or NFTs [8]. They demonstrated that abnormally hyperphosphorylated tau is sufficient to induce neurodegeneration that results in cognitive deficits [12]. Inhibition of the formation of tau hyperphosphorylation could reverse the development of cognitive dysfunction in DM [13].

The ribosomal proteinS6 kinase (S6K) is one of the major downstream targets of mammalian target of rapamycin (mTOR), its phosphorylation level is a well-known parameter to evaluate mTOR activity [14]. mTOR/S6K signalling is required for glucose metabolism and memory formation [15–20]. However, the over-activated mTOR/S6K signalling is thought to be implicated in cognitive dysfunction [21]. The role of mTOR/S6K signalling in the formation of tau hyperphosphorylation has been previously studied in several model systems such as human SH-SY5Y neuroblastoma cells and mouse neuroblastoma 2a (N2a) cells, primary cortical neurons and metabolically active brain slices [22–27]. The abnormal up-regulation of mTOR and S6K was found to be associated with accumulation of hyperphosphorylated tau in AD brain [26, 28]. Inhibition of mTOR with rapamycin improved learning and memory, and reduced tau pathology in a transgenic mouse model of AD [29]. Also, rapamycin treatment improved locomotor and exploratory activity, and prevented neurodenegeration and tau phosphorylation in senescence-accelerated OXYS rats [30]. In our recent study, we found that mTOR/S6K signalling is over-activated in the brains of diabetic mice and that inhibition of mTOR signalling with rapamycin alleviates the phosphorylation level of hyperphosphorylated tau and prevents the formation of DM-induced cognitive deficits [31].

Caveolin-1 (Cav-1), a transmembrane scaffolding protein, found within membrane/lipid rafts, is specifically abundant in neurons in central nervous system [32]. It was reported that neuron-targeted overexpression of Cav-1 enhances functional neuronal membrane/lipid rafts, and improves hippocampus-dependent learning and memory [33, 34]. An accelerated tau-related neurodegeneration and a number of motor and behavioral abnormalities were found in Cav-1 knockout mice [32, 35]. Several studies have demonstrated that Cav-1 negatively regulates mTOR signalling in different cells [36–39], the later one is required for the formation of tau hyperphosphorylation [22, 23]. Thus, we speculate that Cav-1-mTOR/S6K signalling may be involved in the formation of tau hyperphosphorylation. Also, the expression of Cav-1 could be down-regulated in hyperglycemic condition (HGC) in several cells, such as mesenteric vascular smooth muscle cells, lens epithelial cells and monocytes [40–42]. In the hippocampi of STZ-induced diabetic rats that mimic HGC, the down-regulation of Cav-1 was demonstrated by Western blotting [43]. However, how Cav-1 is involved in the formation of tau hyperphosphorylation in HGC is not clear.

Given that DM patients have a higher risk of developing AD in the process of ageing as compared to non-DM control subjects [44], and that neurodenegeration mediated by abnormal tau is implicated in both AD and DM-related cognitive dysfunction [45], it is imperative to elucidate the potential molecular mechanisms underlying the DM induced-neurodegeneration so that to provide appropriate clinical interventions with aims to decrease or remove the risk of developing AD in this DM population. In this study we have mimicked a major biochemical change observed in DM patients: chronic hyperglycemia, in primary hippocampal neurons and STZ-induced diabetic rat model, and have investigated whether or not Cav-1 is involved in the formation of tau hyperphosphorylation in HGC. Our major findings are: 1) that STZ-induced persistent hyperglycemia is associated with cognitive dysfunction, 2) that the up-regulated mTOR/S6K signalling causes the formation of tau hyperphosphorylation in hippocampal neurons, 3) that decreased Cav-1 expression contributes to the formation of tau hyperphosphorylation in HGC, and 4) that mTOR/S6K signalling is required for Cav-1-regulated tau hyperphosphorylation.

## RESULTS

### Correlation of STZ-induced persistent hyperglycemia and cognitive dysfunction

**Figure 1:**
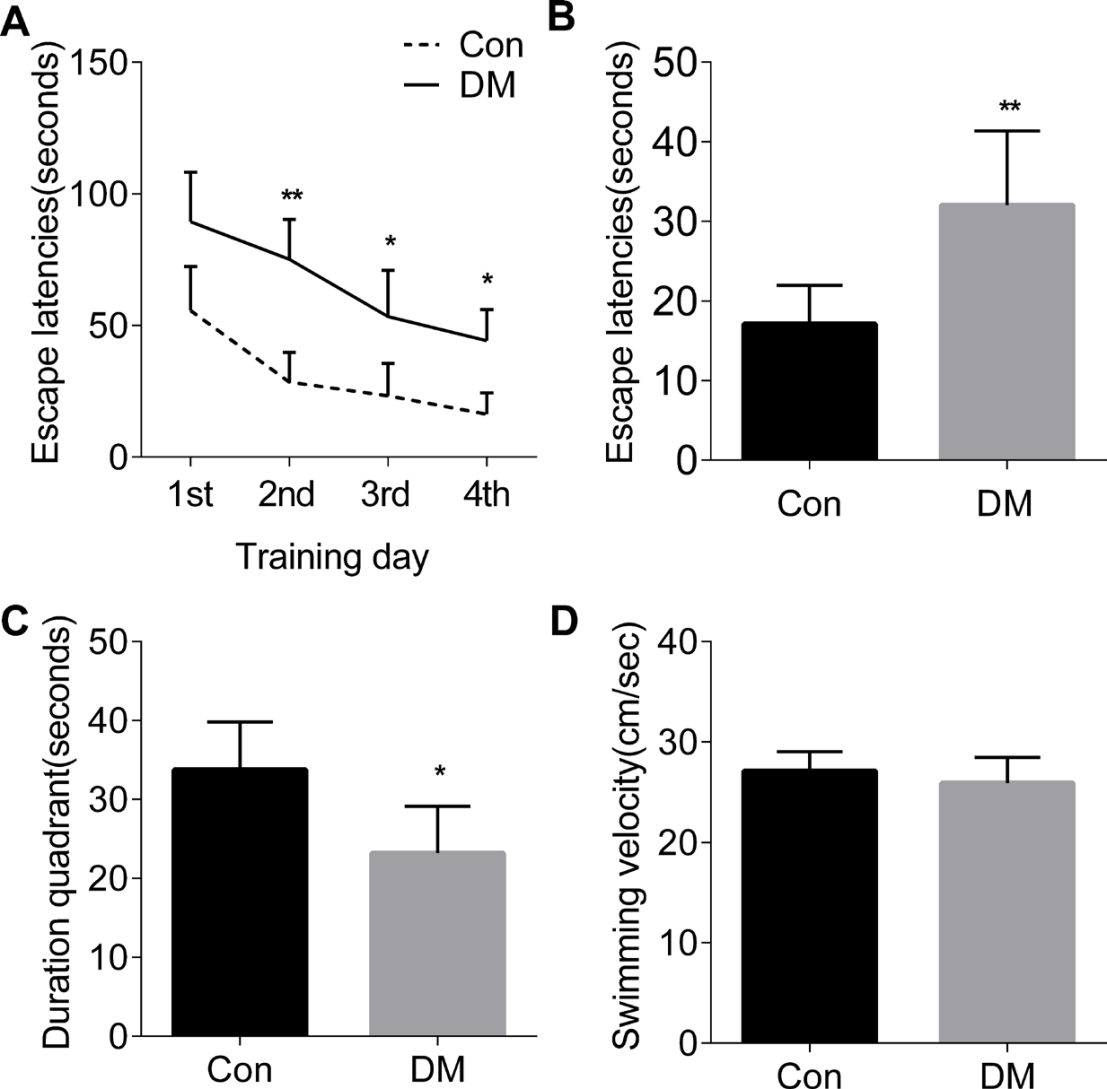
The spatial learning and memory deficits in diabetic rats (**A**) The rat learning acquisition. (**B**) The spatial learning acquistition. (**C**) The spatial memory retrieval. (**D**) The swimming velocity. Con, normal control rats; DM, diabetic rats. *p < 0.05, **p < 0.01 vs Con. Error bars represent s.e.m.

A sharp increase of FBG level was observed from the 3rd day of STZ injection, and the increased FBG level was remained at the 12 th week of STZ injection as compared to the control group (Table 1), suggesting that a persistent hyperglycemia is induced by STZ. In the training, the averaged escape latency of both experimental and control groups decreased in correlation with the progression of the number of training days, and it took more time for the diabetic rats to get on the platform on training day 2, day 3 and day 4 as compared with the control rats (panel Figure 1A). It suggests that a deficit in spatial learning ability is present in diabetic rats. In the tests, diabetic rats showed a significant increase in the escape latency as compared to the controls (panel Figure 1B). In the probe trial, the time spent in the target quadrant with the hidden platform was decreased in diabetic rats compared with the controls (Panel Figure 1C). No significant difference in the swimming velocity was observed between the diabetic and control groups (panel Figure 1D). The ability to escape from a visible platform was also evaluated, and no differences in vision and basal motivation were found in the diabetic and control groups (data not shown). Our results indicated that the acquisition of spatial reference learning and the retrieval of spatial memory are impaired in STZ-induced diabetic rats.

**Table 1:** Summary of experimental groups

Groups	Control group	STZ group
Number of rats	10	10
FBG 3 days (mM)	5.13 ± 0.93	26.13 ± 4.12**
FBG 12 weeks (mM)	5.43 ± 0.62	19.16 ± 1.33**
Body weight 12 weeks (g)	375.28 ± 30.33	280.16 ± 28.91*

STZ and FBG are abbreviations of streptozotocin and fasting blood glucose respectively. All results are presented as mean ± s.e.m. *p < 0.05 vs control group; **p < 0.01 vs control group.

### The up-regulated mTOR/S6K signalling causes the formation of tau hyperphosphorylation in hippocampal neurons

**Figure 2:**
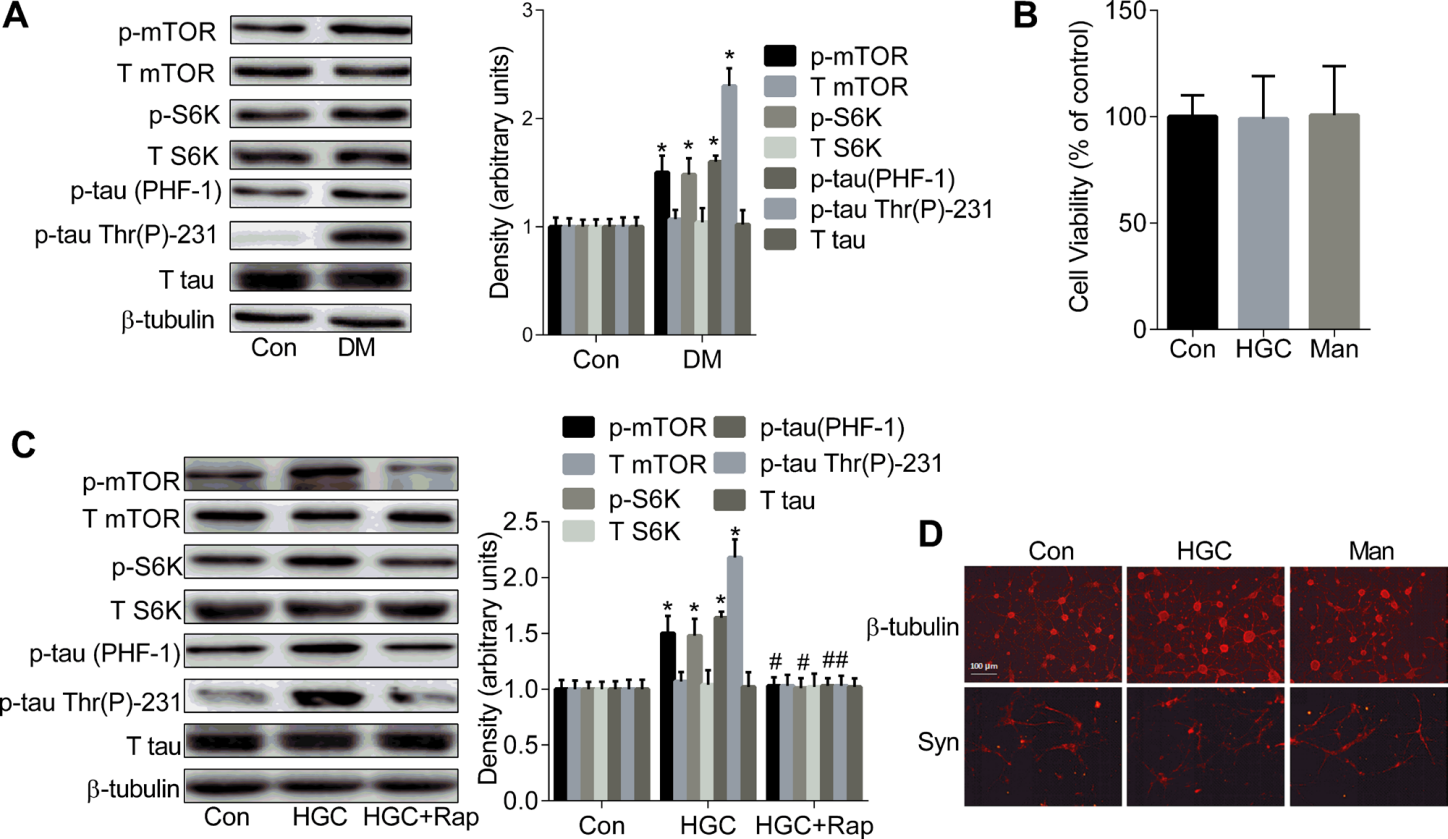
The up-regulated mTOR/S6K signalling causes the formation of tau hyperphosphorylation (**A**) Western blot analysis showed the expression levels of tau phosphorylation and the phosphorylation of mTOR and S6K in the hippocampi of control and diabetic rats brains. (**B**) Cell viability was assessed by CCK-8 assay. (**C**) Western blot showed the levels of tau phosphorylation and the phosphorylation of mTOR and S6K in primary hippocampal neurons exposed to 25 mM glucose (Con), 50 mM glucose (HGC) or 50 mM glucose and 200 nM rapamycin (HGC + Rap) for 6 days. (**D**) The morphology of hippocampal neurons was analyzed by immunofluorescence using anti-β-III tubulin and anti-Syn antibodies. *p < 0.05 vs Con; ^#^*p* < 0.05 vs HGC. Error bars represent s.e.m.

The levels of the p-tau at both Thr231 and Ser396/404 (PHF-1) epitopes but not total tau showed a sharp and significant increase in the hippocampi of diabetic rats as compared to controls (panel Figure 2A). The higher levels of p-mTOR and p-S6K but not the total levels of them were also observed in the hippocampi of diabetic rats as compared to the control rats (panel Figure 2A).

The viability of the primary hippocampal neurons assessed by the CCK-8 assay (panel Figure 2B) showed no significant change between the chronic HGC group (long-term exposure to 50 mM glucose for 6 days) and the osmotic control (25 mM mannitol plus 25mM glucose). The levels of p-tau at both Thr231 and PHF-1 epitopes, p-mTOR and p-S6K were remarkably increased in the HGC group as compared with the control, while levels of total tau, mTOR and S6K remained unchanged (panel Figure 2C), which was reversed by the pretreatment of primary hippocampal neurons with 200 nM rapamycin (panel Figure 2C). However, no dramatic morphological changes were observed in the HGC group as compared to the control by immunofluorescence using anti-neuro-specific class 3β-tubulin and anti-Syn antibodies as neuronal biomarkers (panel Figure 2D).

### Decreased Cav-1 expression contributes to the formation of tau hyperphosphorylation under HGC

**Figure 3:**
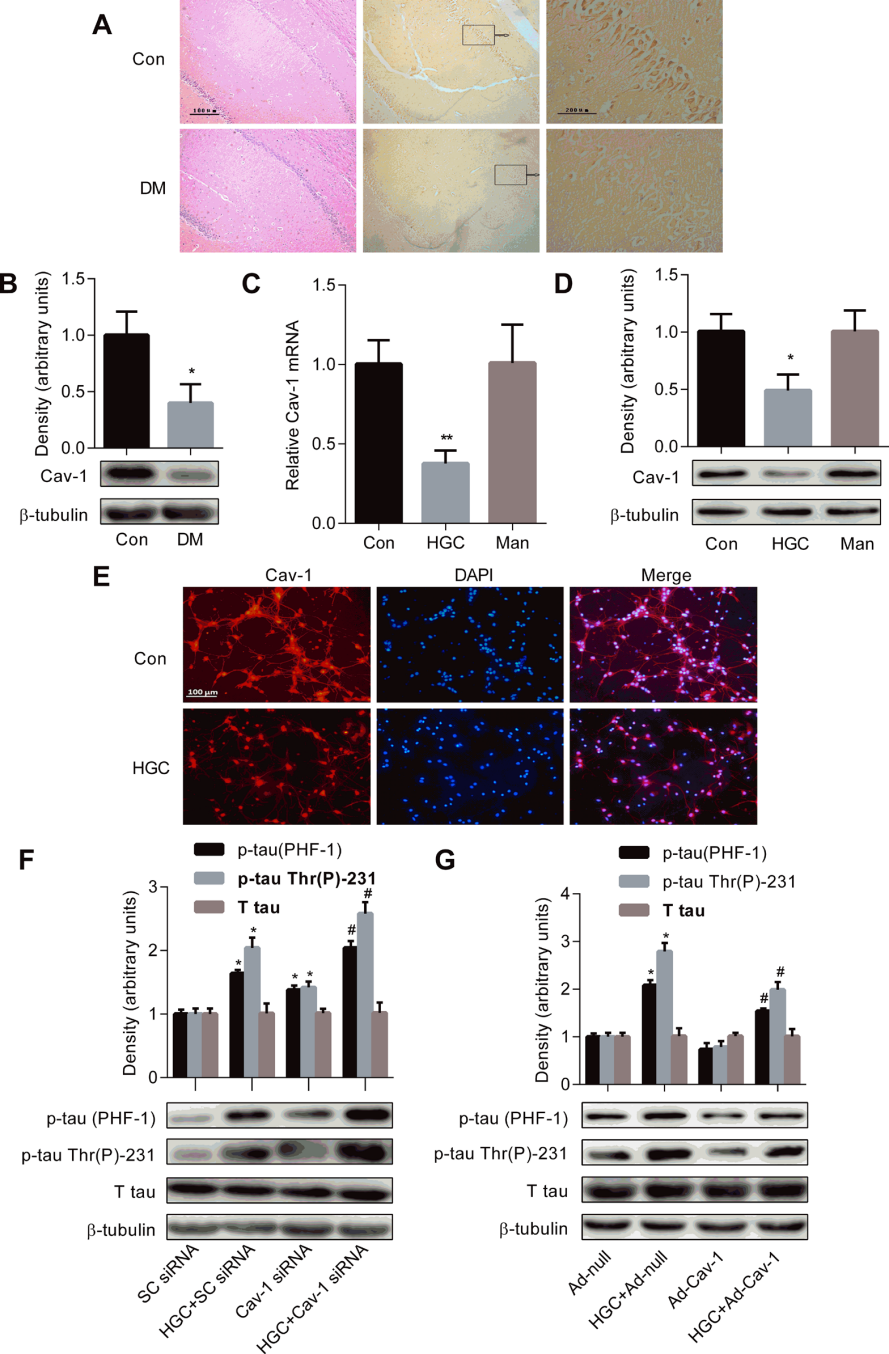
Decreased Cav-1 expression contributes to the formation of tau hyperphosphorylation under hyperglycemic condition (**A**) Representative pictures from paraffin-embedded sections of control and diabetic rats immunostained with antibody to Cav-1. (**B**) Western blot showed the expression level of Cav-1 in the hippocampi of control and diabetic rats brains. Real time PCR (**C**) and Western blot (**D**) showed the expression level of Cav-1 in cultured hippocampal neurons exposed to 25 mM glucose (Con), 50 mM glucose (HGC) or 25 mM mannitol (Man). *p < 0.05, **p < 0.01 vs Con. (**E**) Immunofluorescence staining was used to detect the expression of Cav-1 in hippocampal neurons. Cav-1 was shown in red. (**F**) Knockdown of Cav-1 aggravates tau hyperphosphorylation after HGC. *p < 0.05 vs SC-siRNA; ^#^*p* < 0.05 vs HGC+SC-siRNA. (**G**) Over-expression of Cav-1 ameliorates HGC-induced tau hyperphosphorylation. *p < 0.05 vs Ad-null; ^#^*p* < 0.05 vs HGC + Ad-null. Error bars represent s.e.m.

As shown in panel Figure 3A, faint immunoreactivities of Cav-1 were observed in the CA1 pyramidal neurons of the hippocampus in the diabetic brains, as compared to the control. The protein expression level of Cav-1 by Western blotting was significantly decreased in the hippocampi of diabetic rats (panel Figure 3B).

The mRNA expression of Cav-1 measured by Real time PCR showed a dramatic decrease (62.5%) in 50 mM glucose treated group as compared with control (panel Figure 3C). The protein expression of Cav-1 by Western blots demonstrated 51% reduction in experimental group as compared with control (panel Figure 3D). However, no change was observed in mannitol-treated osmotic control group (panel Figure 3C–3D). By immunofluorescence staining, decreased Cav-1 immunosignals were observed in HG-treated group compared with the control group (panel Figure 3E).

The primary hippocampal neurons transfected with Cav-1 siRNA generated a 77% and 56% reduction for mRNA and protein level, respectively, in the silenced group as compared with the control, whereas scrambled siRNA (SC-siRNA) showed no effect on both protein and mRNA levels of the Cav-1 (Supplementary Figure 1A–1B). As illustrated in panel Figure 3F, in normal condition, transfection of the primary hippocampal neurons with Cav-1 siRNA induced a slight but significant increased levels of p-tau at Thr 231 and PHF-1 epitopes as compared with the SC-siRNA group. In HGC, Cav-1 silenced group showed aggravated levels of p-tau at Thr 231 and Ser 396 / 404 epitopes by 26.5% and 24.4% respectively.

Furthermore, as shown in panel Figure 3G, infection of primary hippocampal neurons with adenovirus encoding Cav-1 showed a dramatic reduction of the levels of p-tau at Thr 231 and PHF-1 epitopes induced by HGC as compared to the control. The over-expression of the Cav-1 was confirmed by both Real time PCR and Western blot methods (Supplementary Figure 2A–2B). These results suggest that Cav-1 is sufficient to reverse HGC-induced tau hyperphosphorylation in hippocampal neurons.

### mTOR/S6K signalling is required for Cav-1-regulated hyperphosphorylation of tau

**Figure 4:**
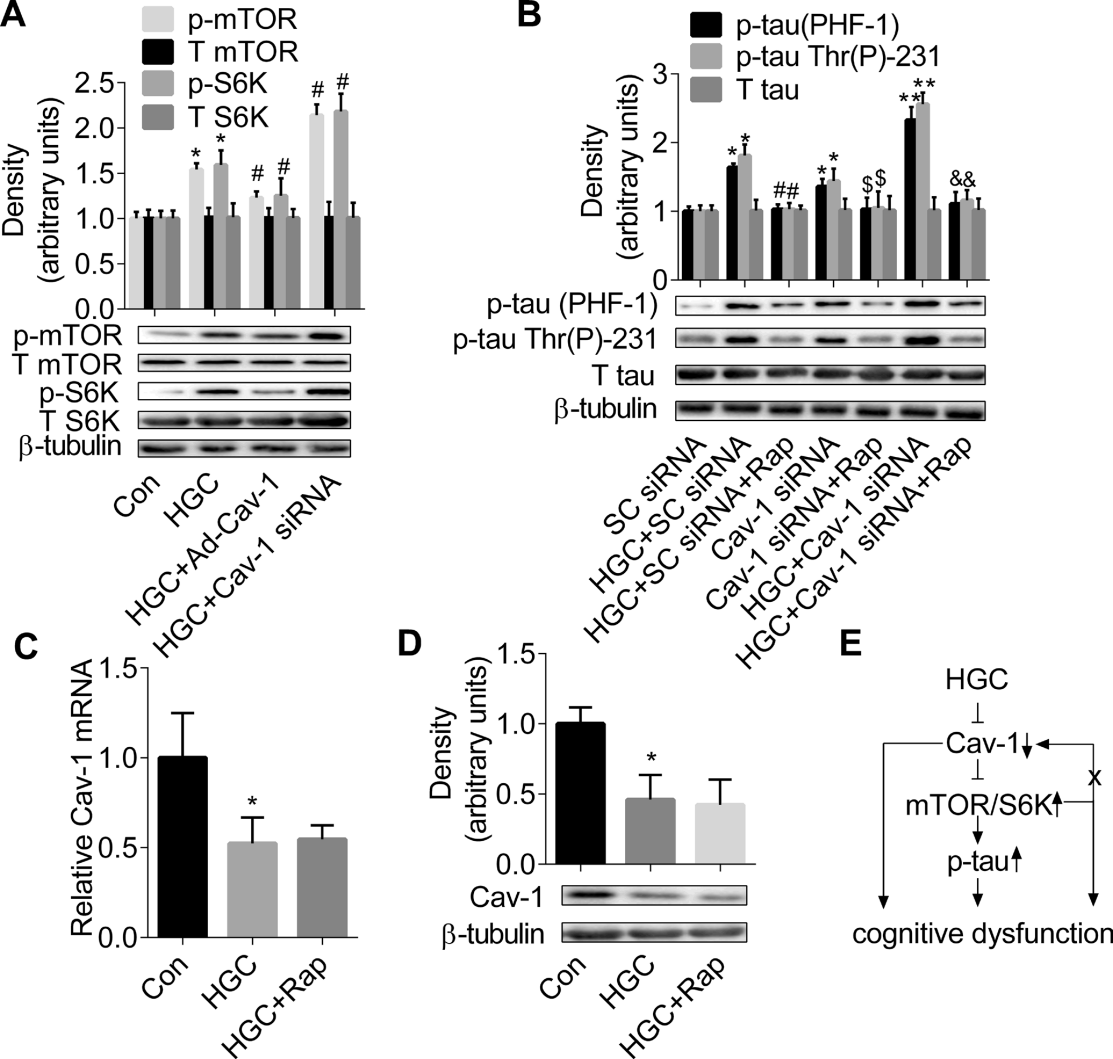
mTOR/S6K signalling is required for formation of Cav-1-regulated tau hyperphosphorylation (**A**) Cav-1-induced counteraction of HGC is associated with mTOR/p70S6K signalling in hippocampal neurons. *p < 0.05 vs Con; ^#^*p* < 0.05 vs HGC. (**B**) mTOR/p70S6K signalling is required for HGC and/or Cav-1 knockdown-induced tau hyperphosphorylation. *p < 0.05 vs SC-siRNA; ^#^*p* < 0.05 vs HGC + SC-siRNA; ^$^*p* < 0.05 vs Cav-1 siRNA; ^&^*p* < 0.05 vs HGC + Cav-1 siRNA. (**C**–**D**) The mRNA and protein expressions of Cav-1 were not regulated by mTOR signalling in hipocampal neurons. *p < 0.05 vs Con. (**E**) a summary of the roles of Cav-1-mTOR/S6K signalling and tau hyperphosphorylation in DM-mediated cognitive dysfunction. Error bars represent s.e.m.

As shown in panel Figure 4, primary hippocampal neurons transfected with Cav-1 siRNA showed much higher levels of p-mTOR and p-S6K in HGC. In contrast, primary hippocampal neurons with Cav-1 overexpression showed a lower level of p-mTOR and p-S6K compared with the HGC group (panel Figure 4A). When neurons were pretreated with mTOR inhibitor rapamycin, tau hyperphosphorylation induced by HG and/or Cav-1 knockdown disappeared (panel Figure 4B). These results suggested that Cav-1 negatively regulates the activation of mTOR/S6K signalling pathway, which is required for HG and/or Cav-1 knockdown-induced tau hyperphosphorylation. Furthermore, as shown in panels Figure 4C–4D, the levels of both Cav-1 mRNA and protein were not affected by rapamycin treatment compared with the HGC group, suggesting the expression of Cav-1 is not regulated by mTOR signalling (Figure 4C–4D).

## DISCUSSION

Chronic hyperglycemia is thought to reduce the function efficiency of brain networks in DM patients, leading to cognitive impairment [46]. In AD APP/PS1 transgenic mice, chronic hyperglycemia was reported to worsen the neuropathological lesion, suggesting that glycemic control may be beneficial for decreasing the incidence of AD in diabetic patients [47]. In the diabetic rats in the present study, STZ-induced persistent hyperglycemia is associated with cognitive dysfunction. The positive correlation between persistent hyperglycemia and cognitive dysfunction was also found in STZ-induced diabetic mice and spontaneously diabetic Goto-Kakizaki (GK) rats [31, 48]. Several DM hyperglycemia ameliorating drugs such as the peroxisome proliferator-activated receptor gamma (PPARγ) agonist, glucagon-like peptide-1 (GLP-1) and dipeptidylpeptidase-4 inhibitor were reported to improve the cognitive deficits in DM patients [49–51]. These results suggested that persistent hyperglycemia maybe one of the primary triggers for the development of cognitive impairment in diabetic patients.

Growing evidence has indicated that neurodegeneration mediated by the formation of hyperphosphorylated tau contributes to the diabetes-associated cognitive deficit [52–54]. Although tau aggregation or intra-cytoplasmic tau-positive tangle-like inclusions were not detected in both type 1 and type 2 diabetes brains [55, 56], different tau phosphorylation sites were reported in the brains of DM patients. Clodfelder-Miller et al. found a rapid and massive increase of tau phosphorylation at multiple residues including Thr 181, Ser 199, Ser 202, Thr 211, Thr 231, Ser 262, and Ser 396 / 404 in the hippocampi of mice for 3 days after STZ treatment [56]. In another mouse study, the change of tau phosphorylations at AT8 (Ser 202 and Thr 205) and PHF-1 (Ser 396/404) epitopes in response to STZ treatment were found to be biphasic, a mild increased tau hyperphosphorylation was observed for 10, 20, and 30 days after STZ injection, and a massive tau hyperphosphorylation was detected after 40 days [53]. In our previous studies, tau hyperphosphorylated at Ser 199 / Thr 202 or Ser 396 site was detected in STZ-induced diabetic mice after STZ-injection for 4 weeks or 45 days, respectively [31, 57]. In the diabetic rats in the present study, in addition to the formation of tau hyperphosphorylated at both Thr 231 and PHF-1 epitopes in the hippocampi, the degree of tau hyperphosphorylation was positively correlated with that of plasma glucose levels and the degree of cognitive dysfunction.

Glucose concentrations larger than 25 mM are considered as HGC that has been used to investigate the potential effects of hyperglycemia in neurons [58]. It was reported that primary hippocampal neuronsexposed for HGC (75 mM) for 24 h induces an increase in tau phosphorylation at Ser 199 [59]. It is believed that, compared withthe *in vitro* short-term (lesser than 24 h) HGC exposure, the long-term (longer than 24 h) HGC exposure is closer to *in vivo* chronic hyperglycemia [60]. Thus, the primary hippocampal neurons isolated from rat brains were exposed to 50 mM HGC for 6 days in the present study. We confirmed that long term HGC (50 mM, for 6 days) significantly increases the levels of tau phosphorylation at both Thr231 and PHF-1 epitopesin primary neurons. Consistent to the results published in a previous study in primary neurons (long term HGC: 50 mM for 7 days) [60], we found that at the similar experimental condition, the morphology and viability of primary neurons are hardly changed, although darkly stained cytoplasm and large cell body were observed in STZ-induced diabetic mice [57]. Probably, *in vitro* HGC exposure for 6 days is not long enough to cause significant cell death but enough to induce the formation of tau hyperphosphorylation. We speculated that inhibiting the formation of hyperphosphorylated tau at the early period of DM may prevent the development of a widespread neuronal degeneration.

As mentioned above, mTOR signalling is required for the formation of tau hyperphosphorylation in several cellular and animal models such as human SH-SY5Y neuroblastoma cells and mouse neuroblastoma 2a (N2a) cells, primary cortical neurons, metabolically active brain slices, triple-transgenic model of AD (3xTg-AD) mice and tau P301S transgenic mice [22–27, 61, 62]. mTOR/S6K signalling could regulate glucose metabolism including inhibiting glucose uptake, improving glycogen synthesis and stimulating glycolysis [15–19], and a feedback regulation mechanism of mTOR by glucose metabolism is suggested by the findings that HGC activates mTOR signalling in rat PC12 pheochromocytoma cells (a clonal cell line closely related to sympathetic neurons) and HT22 cells (an immortalized mouse hippocampal cell line) [63, 64]. It was demonstrated that the chronic activation of mTOR by high glucose results in insulin resistance [65] and the hyperactivation of mTOR is involved in diabetic complications [66]. We found sharp increased levels of p-mTOR and p-S6K in the hippocampi of STZ-induced diabetic rats, the increases correlates with the levels of p-tau. Increased levels of p-mTOR, p-S6 (the substrate of S6K) and p-tau were also reported in Zucker diabetic fatty rat brains [67]. Recently, we showed that pharmacological inhibition of the over-activated mTOR/S6K signalling with rapamycin rescues cognitive deficits and decreases tau hyperphosphorylation in STZ-induced mouse DM model [31].

The indispensable roles of Cav-1 on neuronal system have been demonstrated in different animal and cell models: improvement of learning and memory in neuron-targeted Cav-1 overexpression mice [34], enhanced brain lesion volume and neuroinflammation in Cav-1 deficient mice [68], accelerated neurodegeneration in Cav-1 knockout mice [35], and a progressive decrease in Cav-1 expression under HGC accelerated the neuregulin-induced degenerationin Schwann cells (SC)/dorsal root ganglion (DRG) co-cultures [69]. Recently, enhanced hepatic gluconeogenesis and glucose output were observed in the Cav-1 null mice [70], and suppressed glycolysis was noted in Cav-1 reconstituted breast cancer epithelial cells [71], suggesting its implication in glucose metabolism. In addition to the evidence that Cav-1 expression is down-regulated by HGC in several cells [40–42, 69], Cav-1 function has also been investigated in diabetic nephropathy [72], diabetic retinopathy [73], and diabetic wound healing [74]. In the present study, a significant decrease of Cav-1 was found by Western blotting in the homogenates prepared from the hippocampi of diabetic rats, which is consistent with the results from the previous study [43]. A decreased Cav-1 immunoreactivity was consistently observed by immunohistochemistry in the hippocampal neurons in the brains of diabetic rats and by immunofluorescence in primary neurons in HGC group. Furthermore, we demonstrated that Cav-1 over-expression protects neurons from formation of tau hyperphosphorylation induced in HGC, whereas silencing Cav-1 expression aggravates the degree of tau hyperphosphorylation. Our results suggested that decreased Cav-1 expression contributes to the formation of tau hyperphosphorylation in HGC. However, an opposite report demonstrated that overexpression of Cav-1 increased ratio of tau-Ser 404/tau protein in N2a/APP695swe cells, which stably expressing APP [75]. This discrepancy might be due to different cell types (primary hippocampal neurons or N2a cell lines) and experimental models (DM-related or AD-related cellular model).

A negative regulation of mTOR signalling by Cav-1 has been demonstrated in several cellular model systems: a remarkable increase of mTOR acitvity in Cav-1-deficient cancer-associated fibroblasts [36], hypoactivation of mTOR signalling in Cav-1-overexpressing U87MG cells [37, 38] inhibited the activation of PI3K/AKT/mTOR signalling in HL-60 cells with Cav-1 over-expression [39]. In the present study, Cav-1 over-expression inhibited HGC-induced increased level of p-mTOR and p-S6K, whereas Cav-1 silencing aggravated the over-activated mTOR/S6K signalling. Our results for the first time in literatures indicated that Cav-1negatively regulates the activity of mTOR/S6K signalling pathway in neurons. However, we have also noticed that a positive correlation between Cav-1 and mTOR signalling is reported in literatures, such as a sustained activation of Cav-1 and PI3K/Akt/mTOR signalling cascades by low shear stress in breast carcinoma MDA-MB-231 cells [76], and galectin-1-induced p-mTOR inhibition by Cav-1 siRNA in mouse embryonic stem cells [77]. This discrepancy might be due to different cell types and different treatment factors. Our findings that the inhibition of mTOR with rapamycin attenuated Cav-1 deficiency-induced tau hyperphosphorylation suggest that Cav-1 negatively regulates tau phosphorylation via mTOR/S6K signalling pathway. However, neither mRNA or protein expression of Cav-1 was affected by rapamycin treatment. These evidence suggest that a sustained over-activation of mTOR signalling induced by Cav-1 down-regulation can not form a negative feedback loop to reverse the abnormal expression of Cav-1.

In physiological condition, mTOR plays a role in memory processing [20]. Taken together, it suggests that the decline of cognitive function in DM is triggered by hyperglycemia, the process of cognitive dysfunction is contributed by suppressed Cav-1 expression, over-activated mTOR/S6K signalling, and/or the formation of tau hyperphosphorylation (panel Figure 4E). Although this mechanism is needed to be further confirmed in DM patients, the results available so far should encourage more studies to explore the protective effects of Cav-1 in cognitive dysfunction. Our results at least suggest a potential pharmaceutical intervention approach in diabetes-induced cognitive dysfunction.

## MATERIALS AND METHODS

### Antibodies and materials

Antibodies used in the study include: rabbit monoclonal anti-β III tubulin for Western blot analysis (Abcam; ab52901; 1/8,000); rabbit monoclonal anti-PHF1 (Abcam; ab184951; 1/5,000); rabbit monoclonal anti-tau (phospho T231) (Abcam; ab151559; 1/5,000); chickenanti-tau (Abcam; ab75714; 1/30000); rabbit monoclonal anti-mTOR (phospho S2448) (Abcam; ab109268; 1/5,000); rabbit anti-mTOR (Cell Signalling; 2983; 1/1,000); rabbit anti-Phospho-S6K (Thr421/Ser424) (Cell Signalling; 9204; 1/1,000); rabbit anti-S6K (Cell Signalling; 9202; 1/1,000); rabbit anti-Cav-1 for western blot analysis (Santa Cruz Biotechnology; sc-894; 1/500); rabbit anti-Cav-1 for immunohistochemical staining (Santa Cruz Biotechnology; sc-894; 1/100); rabbit anti-Cav-1 for immunofluorescence (Santa Cruz Biotechnology; sc-894; 1/50); rabbit monoclonal anti-Synaptophysin (Syn) (Abcam; ab32127; 1/50); rabbit monoclonal anti-β III tubulin for immunofluorescence (Abcam; ab52901; 1/100).

The adenoviral Cav-1 (Ad-Cav-1) and the empty adenovirus vector (Ad-null) were generously gifted from Dr. Duan-Fang Liao (Hunan University of Traditional Chinese Medicine, China).

### Characterization of STZ-induced diabetic rats

Male Sprague-Dawley (SD) rats (weighing 190–220g) were purchased from the Experimental Animal Center of Central South University, Hunan, China. All experimental procedures were conducted according to the strict guidelines of the Animal Welfare Committee of Central South University. STZ (60 mg/kg) was intraperitoneally injected into rats to induce HGC observed in DM patients as previously described [78]. The control groups were injected with an equivalent volume of citrate buffer. Fasting plasma glucose (FPG) level was measured by the glucose oxidase method (GOD-PAP; Boehringer-Mannheim). When the FPG level of rats is higher than 300 mg/dl (*n* = 10) 3 days after STZ injection, diabetes was diagnosed. Behavioral experiments were carried out and rats were sacrificed in the 12th week. Since the hippocampus plays a central role in regulation of spatial learning and memory in rodents [79], hippocampal tissues in diabetic rats from hippocampi were selectively used to investigate the mechanism of cognitive impairment induced by hyperglycemia. Five rats each group were sacrificed and usedin immunohistochemistry and Western blot analyses.

### Cognitive measurement by Morris water maze test

The abilities of spatial learning and memory were measured in diabetic and control rats by the Morris water maze test as previously described [80] prior to be sacrificed. A circular pool (250 cm in diameter) filled with water at 24°C–26°C was divided into four quadrants. For training, a submerged platform (15 cm in diameter) was fixed in the center of the fourth quadrant. Rat behaviors (latency, distance, swim speed, and navigation path) were recorded with a video camera connected to a computerized tracking system. The spatial learning ability was assessed by the escape time to reach the platform (escape latency). 24 h after testing trials, the ability of spatial memory was evaluated by the time spent in the target quadrant in which no platform was placed.

### HGC, infection, transfection, and rapamycin treatment in primary hippocampal neurons

Primary hippocampal neurons were prepared as described by Wang et al. [81]. Briefly, after papain digestion, hippocampal neurons isolated from 18-day-old SD rat embryos were seeded on plates coated with 0.1mg/ml poly-D-lysine in Dulbecco's Modified Eagle Medium/Nutrient Mixture F-12 (DMEM/F12) (Gibco Life Technologies, USA) containing 10% FBS in a 37°C humidified incubator with 5% CO_2_ for 4 h. The seeded hippocampal neurons were cultured with Neurobasal medium (Gibco Life Technologies, USA), supplemented with B27 (1:50 dilution; Gibco Life Technologies, USA). Half of the medium was replaced every 2–3 days. After 6 days, the experiments were carried out as following:

### HGC

The primary neurons were incubated with 50 mM glucose (HGC group) or 25 mM mannitol (plus 25 mM glucose in Neurobasal medium, control group), and maintained for another 6 days.

### Viral infection

The primary neurons were infected by Ad-Cav-1 or Ad-null. To confirm the over-expression of Cav-1, the neurons were harvested at 24 h for RNA extraction and at 48h for protein extraction after infection. After 48 h infection, the infected primary neurons were exposed to HGC for another 6 days in order to study the potential effect of Cav-1 over-expression on HGC-induced tau hyperphosphorylation.

### Transfection

The primary neurons were transfected with Cav-1 siRNA or scrambled control siRNA (SC-siRNA) (Invitrogen, USA) using Lipofectamine RNAi MAX (Invitrogen, USA) following the manufacturer's instructions. In order to confirm the silencing degree of Cav-1, the neurons were harvested at 24 h for RNA extraction and at 48h for protein extraction after tranfection. After 48 h transfection, the transfected neurons were exposed to HGC for another 6 days for studying the potential effect of Cav-1 knockdown on HGC-induced tau hyperphosphorylation.

### Rapamycin treatment

the transfected and non-transfected primary neurons were pretreated with rapamycin (200 nM; Sigma, USA) or DMSO for 2 h prior to the exposure of HGC for studying the potential role of mTOR signalling in HGC-inducedtau hyperphosphorylation.

### Neuronal viability assay

The primary neurons were plated in 96 well plates and cultured for 6 days, and half of the medium was replaced every 2–3 days. After exposed to HGC for another 6 days, the neuronal viability was determined following the protocol provided for the Cell Counting Kit-8 (CCK-8) kit (Dojindo, Japan). Briefly, culture media were absorbed and replaced with 10% CCK-8in Neurobasal/B27 medium. After incubation at 37°C for 4 h, absorbance was measured at the wavelength of 450 nm with 650 nm as reference.

### RNA extraction and RNA level determination

After experiments, total RNA was isolated from primary hippocampal neurons using TRIzol reagent (Invitrogen, USA) as described previously [82]. The levels of mRNA for Cav-1 and β-actin were analyzed by realtime quantitative RT-PCR using SYBR-Green dye and Applied Biosystems (GeneCopoeia, USA). The mRNA for Cav-1 was amplified and quantified with primers as listed below. The synthetic oligonucleotide primer sequences for Cav-1 and β-actin were as follows: Cav-1 5′-TCTACAAGCCCAACAACAAGGCC-3′ (upstream) and 5′-TGCACTGAATCTCAATCA-GGAAGC-3′ (downstream); β-actin 5^′^-CAACGAGCGGTT- CCGAT-3′ (upstream) and 5′-GCCACAGGATTCCATACCCA-3′ (dowmstream). Amplification condition was set as follows: pre-denaturation at 95°C for 5min, 40 cycles of denaturation at 95°C for 10 s, annealing at 60°C for 20s, extension at 72°C for30s. Cav-1 mRNA levels were normalized to that of β-actin.

### Protein extraction, protein measurement and Western blot analysis

After behavioral experiments were carried out, five rats each group were sacrificed under deep anesthesia with diethylether. Hippocampi were dissected from rat brains and homogenized in buffer containing 50 mM Tris, pH 7.0, 2.5 mM EDTA, 2.5 mM EGTA, 2 mM benzamidine, 0.5 mM PMSF, 0.1% β-mercaptoethanol, 20 mM β-glycerophosphate, 0.1% protease inhibit or cocktail (Sigma, USA), 2 mM sodium vanadate, 50 mM NaF, and 2% SDS at 4 °C [83]. Protein concentration was detected with a bicin choninic acid (BCA) protein assay kit (Beyotime, China).

Primary neurons were harvested and lysed in iced lysis buffer containing 40 mM HEPES, 120 mM NaCl, 1 mM EDTA, 10 mM pyrophosphate, 10 mM glycerophosphate, 50 mM NaF, 0.5 mM or thovanadate, 1% Triton X-100, and 1% protease inhibitor mixture [23]. The supernatant samples were obtained after centrifuging at 13,000 × g for 20 min. Twenty microgram protein samples were loaded each lane. Protein samples were separated by SDS-polyacrylamide gels (SDS-PAGE) and transferred to nitrocellulose membrane. The membranes were blocked with 5% non-fat milk prior to incubation with primary antibody. Membranes were washed with TBS-T (Tris-buffered saline supplemented with 0.1% Tween-20) and incubated with secondary antibodies (ZSGB-BIO, China). Relative density of each protein band was normalized to that of β-III tubulin. All results were representative of at least five independent experiments.

### Immunofluorescence and immunohistochemsitry

The primary hippocampal neurons were plated on poly-l-lysine coated 22 mm diameter glass coverslips at a density of 2×10^5^ cells per coverslip and cultured for 6 days, and half of the medium was replaced every 2–3 days. After the neurons were exposed to HGC for another 6 days, the coverslips were removed from the wells and placed in coverslip holders. Then the neurons were fixed with 4% paraformaldehyde and permeabilized with 0.1% Triton X-100.After 10 min, the neurons were blocked with 1% bovine serum albumin (BSA) (Sigma, USA)at room temperature for 20 min. After washing, the neurons were incubated with primary antibodies at 4°C overnight, followed by incubation with FITC-conjugated secondary antibodies. The slides were mounted and observed under fluorescence microscope (Leica, PA).

After behavioral experiments were carried out, five rats each group were sacrificed under deep anesthesia with diethyl ether and transcardially perfused with 4% paraformaldehyde pH 7.4, and whole brain was dissected and embedded in paraffin according to the method described in our previous publication [83]. The tissue blocks were sectioned in 6 μm thickness. To eliminate non-specific binding, the sections were treated with 3% normal goat serum at room temperature. The sections were then incubated with at primary antibodies at 4°C overnight, followed by incubation with biotinylated secondary antibody room temperature for 2 h, and visualized using the avidin-biotin-peroxidase complex kit (ZSGB-BIO, China) with 3–3′-diaminobenzidine-4 HCl/H_2_O_2_ (DAB; Sigma) as a substrate.

### Statistical analysis

The data were expressed as the mean ± s.e.m. For the comparison of means between two groups, student's *t* test was used. For the comparison of means ≥ three groups, one-way ANOVA and the Tukey's test were used. *p* ≤ 0.05 were considered statistically significant.

## SUPPLEMENTARY MATERIALS FIGURES


