# Supplementary material for: High glucose induces formation of tau hyperphosphorylation via Cav-1-mTOR pathway: A potential molecular mechanism for diabetes-induced cognitive dysfunction
Source: Oncotarget. 2017 Apr 19;8(25):40843–56. doi: 10.18632/oncotarget.17257 (PMC5522306; doi:10.18632/oncotarget.17257)
Supplement: Supplementary file 1 [file oncotarget-08-40843-s001.pdf]

High glucose induces formation of tau hyperphosphorylation via Cav-1-mTOR pathway: A potential molecular mechanism for diabetes-induced cognitive dysfunction

Supplementary Materials

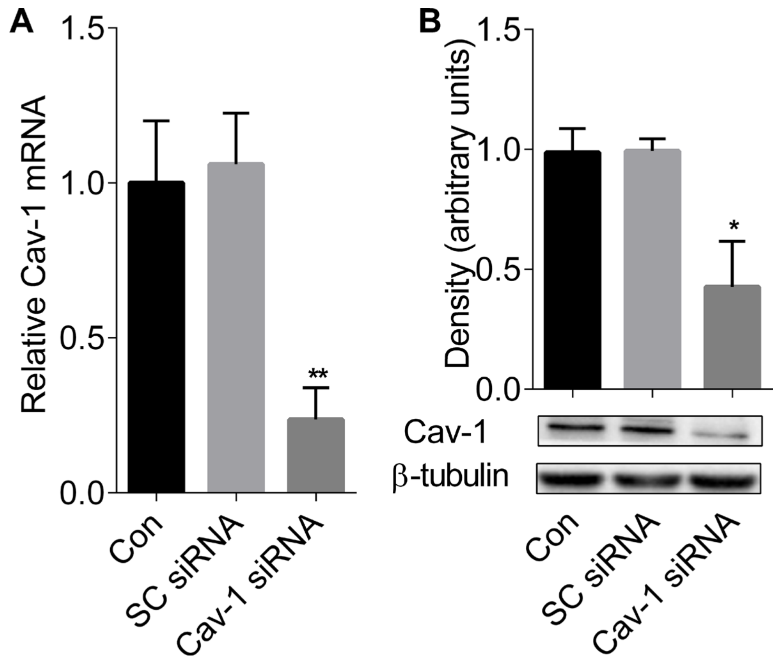

Supplementary Figure 1: The deletion of Cav-1. The expression level of Cav-1 after transfection was determined by Real time PCR (**A**) and Western blot (**B**). $^{*}p < 0.05$, $^{**}p < 0.01$ vs SC- siRNA. Error bars represent s.e.m.

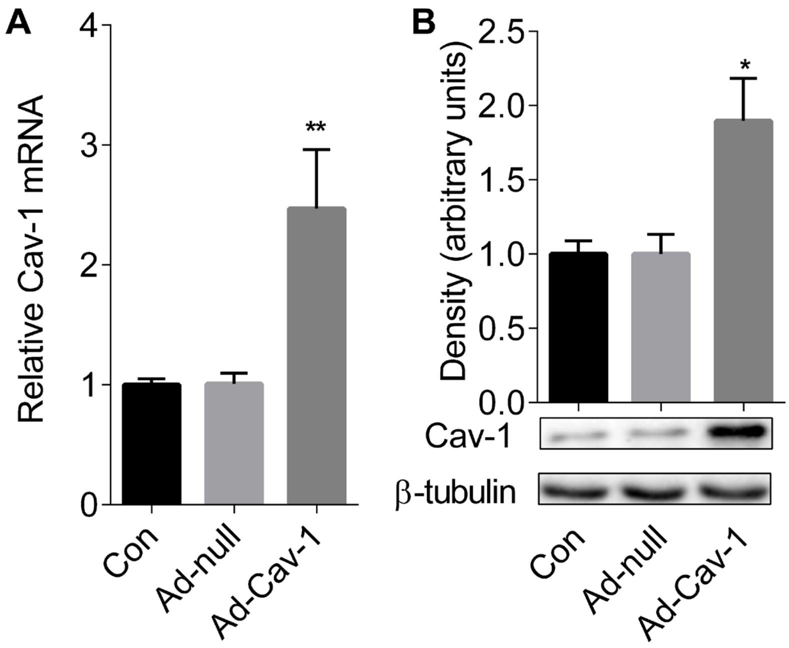

Supplementary Figure 2: The overexpression of Cav-1. The expression level of Cav-1 after transfection was determined by real time PCR (**A**) and Western blot (**B**). *$p < 0.05$, **$p < 0.01$ vs Ad-null. Error bars represent s.e.m.
